# MECHANICAL AND STRAIN BEHAVIOUR OF HUMAN ACHILLES TENDON DURING *IN VITRO* TESTING TO FAILURE

**DOI:** 10.22203/eCM.v043a12

**Published:** 2022-04-21

**Authors:** C.V. Nagelli, A. Hooke, N. Quirk, C.L. De Padilla, T.E. Hewett, M. van Griensven, M. Coenen, L. Berglund, C.H. Evans, S.A. Müller

**Affiliations:** 1 Rehabilitation Medicine Research Center, Mayo Clinic, Rochester, MN, USA; 2 Department of Orthopaedic Surgery, Mayo Clinic, Rochester, MN, USA; 3 Department of Cell Biology-Inspired Tissue Engineering, MERLN Institute, Maastricht University, the Netherlands; 4 Department of Orthopaedic Surgery, University of Basel, Basel, Switzerland; 5 Hewett Consulting, Minneapolis and Rochester, MN, USA; 6 The Rocky Mountain Consortium for Sports Research, Edwards, CO, USA

**Keywords:** Strain behaviour, Achilles tendon, digital image correlation, tendon rupture

## Abstract

The Achilles tendon is the strongest tendon in the human body but its mechanical behaviour during failure has been little studied and the basis of its high tensile strength has not been elucidated in detail. In the present study, healthy, human, Achilles tendons were loaded to failure in an anatomically authentic fashion while the local deformation and strains were studied in real time, with very high precision, using digital image correlation (DIC). The values determined for the strength of the Achilles tendon were at the high end of those reported in the literature, consistent with the absence of a pre-existing tendinopathy in the samples, as determined by careful gross inspection and histology. Early in the loading cycle, the proximal region of the tendon accumulated high lateral strains while longitudinal strains remained low. However, immediately before rupture, the mid-substance of the Achilles tendon, its weakest part, started to show high longitudinal strains. These new insights advance the understanding of the mechanical behaviour of tendons as they are stretched to failure.

## Introduction

Tendons connect skeletal muscles to bones, enabling movement by transmitting the forces generated by muscle contraction. They comprise highly ordered, hierarchical collagenous structures that withstand large loads when stretched. They are frequently injured during sporting activities and other trauma (Schepsis et al., 2002), and the incidence of tendon ruptures has increased in recent years (Ganestam et al., 2016; Lantto et al., 2015). Because tendon injuries do not heal well (Docheva et al., 2015; Muller et al., 2015) and interfere with quality of life, they create an increasing medical, economic and social burden (Hopkins et al., 2016). A detailed understanding of the mechanical properties of tendons, as well as strain patterns leading to rupture, may provide important new information related to the unique strength of tendons and their mechanism of failure.

**Fig. 1.**
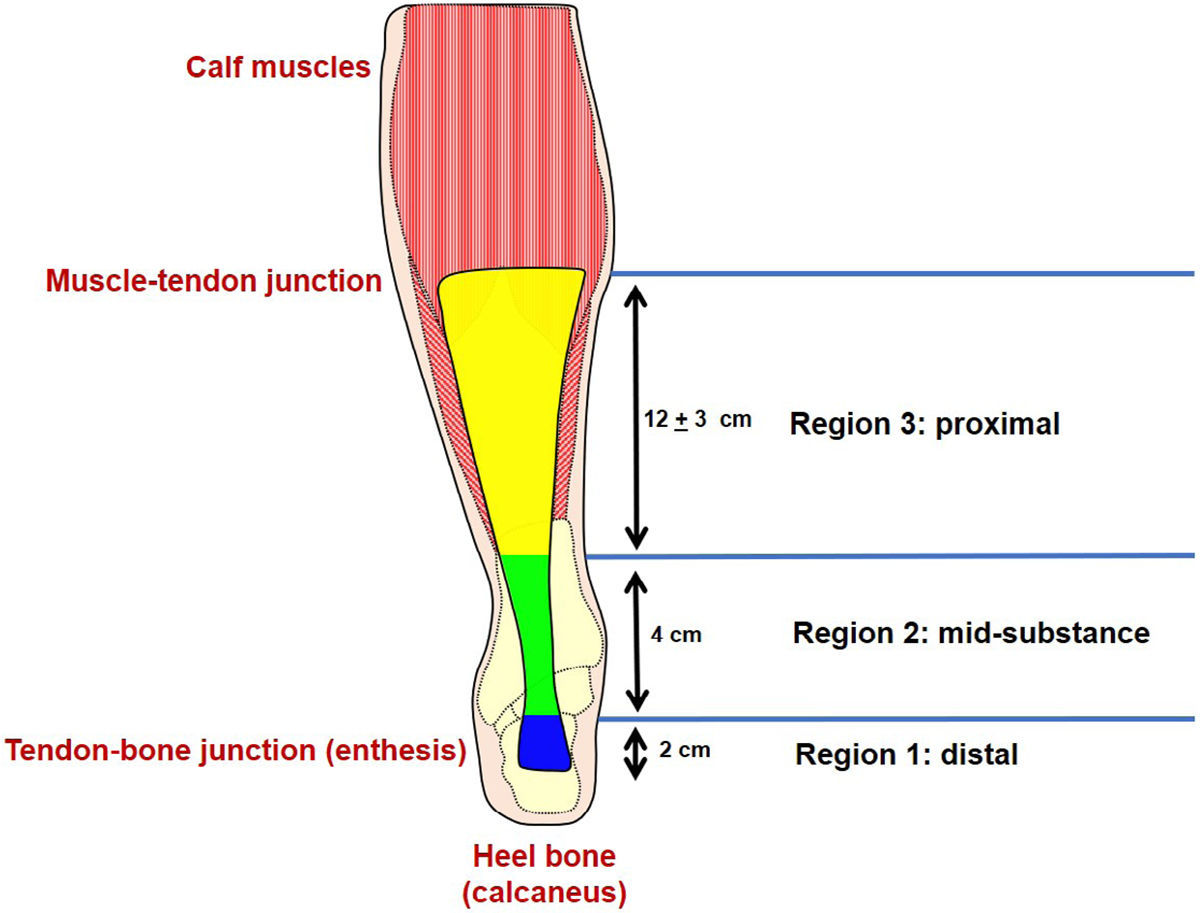
A schematic of the human Achilles tendon, showing division into 3 regions of analysis.

The Achilles tendon connects the calf muscles to the heel (calcaneus; Fig. 1). It is the largest and strongest tendon in the human body and resists forces that exceed 12 times the body weight during simple activities such as running (Komi et al., 1992). However, the basis for the unusual strength of the normal human Achilles tendon is poorly understood. It is often assumed that its strength reflects its large size, but such a simplistic explanation ignores the fact that the Achilles tendon narrows to a width of only 1.8 cm at its mid-substance (Del Buono et al., 2013), where most Achilles tendon ruptures occur.

The lack of information concerning the mechanical properties of the human Achilles tendon results partly from practical and technical challenges encountered when attempting to study this tissue *ex vivo*. The limited availability of normal, fresh, human Achilles tendons is one factor. Another challenge is the ability to fix Achilles tendon for testing in an anatomically authentic fashion, with its calcaneal insertion intact at one end and the calf muscles secured at the other exactly in line with the axis of loading. The magnitude of these constraints is reflected in the dearth of literature on the subject. Only three published papers were identified in which the mechanical properties of freshly excised, normal, human Achilles tendons were measured during loading to failure (Louis-Ugbo et al., 2004; Wren et al., 2003; Wren et al., 2001). One additional study used Achilles tendon removed from embalmed cadavers (Lewis and Shaw, 1997). A particularly severe additional complication is the non-uniform cross section of the Achilles tendon, which renders the stresses and strains highly inhomogeneous along the length of the tendon.

The present study attempted to overcome the limitations of the literature. One of the main objectives was to measure the failure loads of fresh, uninjured, human Achilles tendons from young donors when the tendons are stretched to failure while being aligned in an anatomically authentic fashion. In addition, digital image correlation (DIC) was used to study the strain behaviour of the same tendons. This technology allowed for measuring, for the first time, local deformation strains in real time with high precision during loading to failure. The study hypothesis was that this group of healthy tendons would fail at loads exceeding those reported previously and that it would be possible to gain insight into deformation patterns as the tendons were stretched to failure.

## Materials and Methods

### Sample preparation

**Fig. 2.**
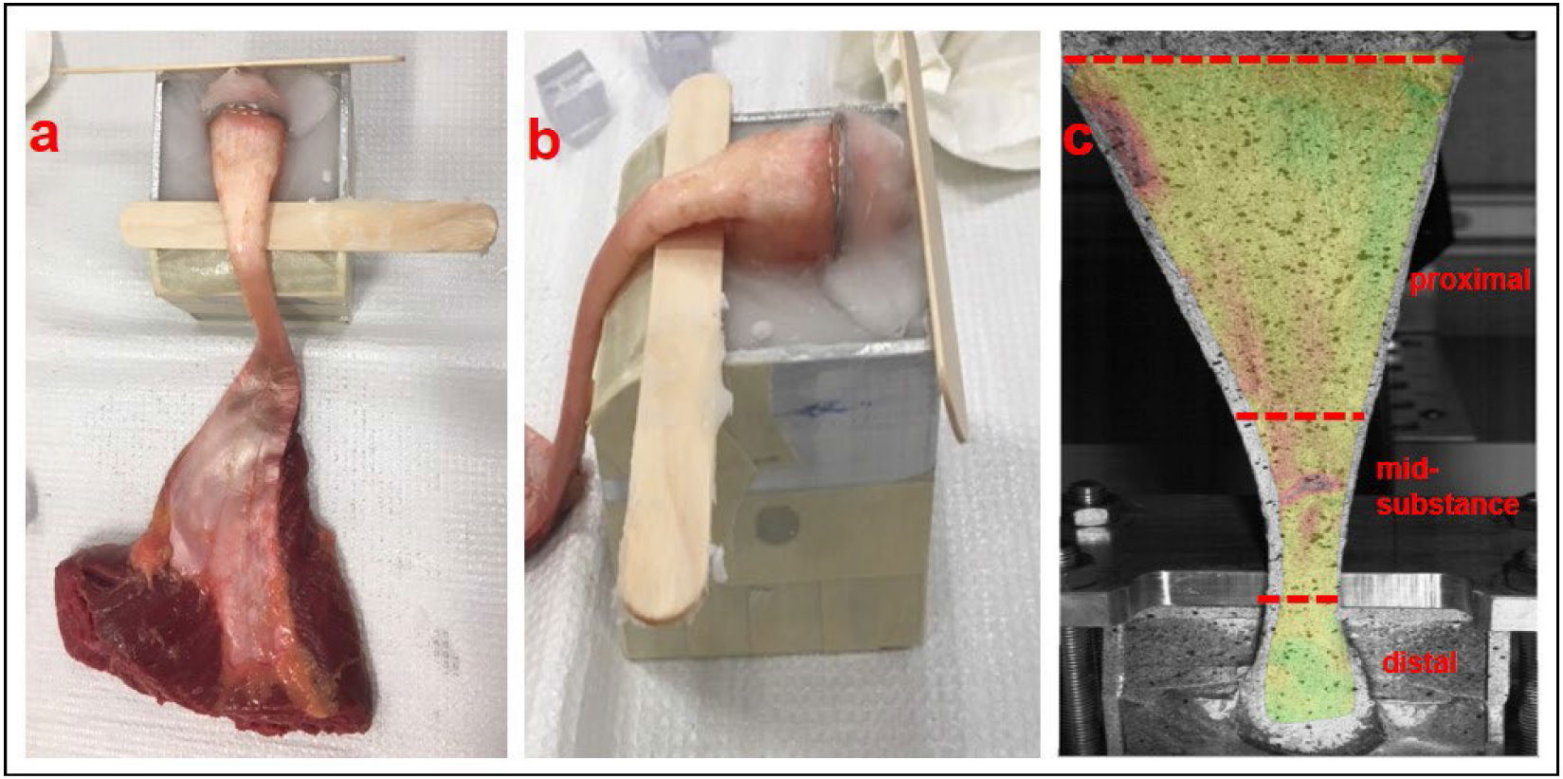
Tendon preparation for mechanical testing. (**a**) Frontal and (**b**) sagittal plane views of the Achilles tendon insertion site, with the calcaneus exposed and secured in a metal block using a bone cement. (**c**) The experimental set-up includes simultaneous tensile testing and local strain measurements using a digital imagine correlation system. The Achilles tendon was in an anatomically correct position, with the calcaneus position at a 30° angle, to simulate the natural position of a plantigrade foot in a neutral position. The mid-substance of the tendon did not have contact with the frame during testing.

Fresh, frozen, complete human cadaveric lower extremities were obtained (Anatomy Gifts Registry, Hanover, MD, USA) from 10 donors (5 male/5 female), age 42.1 ± 9.8 years (height 1.78 ± 0.1 m; weight 83.3 ± 22.3 kg), with no history of traumatic lower extremity injury or surgery. One limb each was used from 9 of the donors; both limbs were used from one male donor, giving a total of 11 Achilles tendons (5 female and 6 male tendons). The entire Achilles tendon, including the calcaneus and distal third of the *gastrocnemius* and *soleus* muscles, was harvested from each donor after which the enthesis was carefully exposed by dissection. A board-certified orthopaedic surgeon (SAM) examined the tendons for macroscopical signs of injury or degeneration. Samples were secured in a metal fixture using bone cement and K-wire wrapping such that a portion of the calcaneus was above the surface, with the enthesis clearly visible (Fig. 2). The samples were kept hydrated during dissection using saline.

### Mechanical testing

Samples were loaded into a servo-hydraulic testing machine (model 312, MTS System Corporation, Eden Prairie, MN, USA) using a customised fixator, with the calcaneus at a 30° angle to simulate the anatomic position of a plantigrade foot in a neutral position. The muscle and musculotendinous junction were fixed using a custom cryo-clamp, which includes two metal plates that are bolted together and have ridges on their inside surface to prevent the muscle from slipping during loading. These plates also include an inlet for CO_2_ (which fills a chamber within the plates) that releases the gas through outlets on the inside, ridged surface that is clamping the tissue. After securely fastening the tissue between the plates, the tissue was exposed to 30 s of CO_2_, which cryoclamped the tissue just before testing. Then, tendons were pre-conditioned under 10 cycles of pre-load to 2 % strain before being subjected to displacement-controlled tensile testing at 5 mm/s until failure. Force was measured using a 2,500 kg load cell (model 3397; Lebow products, Troy, MI, USA; accuracy 0.05 %). Force and displacement data were collected at 128 Hz.

### DIC

**Fig. 3.**
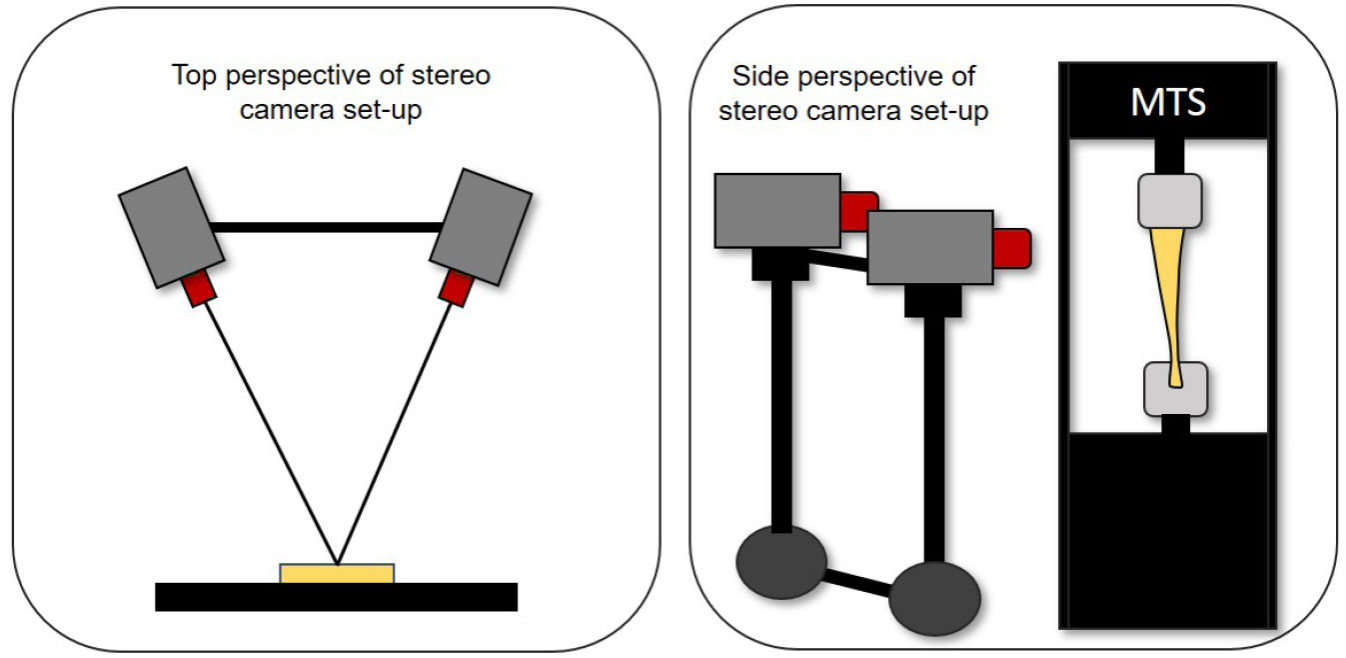
A schematic of the stereo camera set-up for digital image correlation of human Achilles tendons during tensile loading.

For DIC measurements, the posterior surface of each tendon was patterned first with a solid white, mineral-oil-based face paint (Mehron, Inc., Chestnut Ridge, NY, USA), with black speckling applied on top of the white base layer using spray paint. The samples were kept hydrated during testing using saline, which was carefully applied using a spray bottle so as not to distort the speckling pattern. DIC imaging was performed using a GOM-ARAMIS 4M system (GOM-Optical Measuring Techniques, Braunschweig, Germany). The system consists of two cameras that were mounted on opposite sides of a rolling frame and placed about 3 m from the tendons within the MTS machine (Fig. 3). The cameras have a resolution of 4 megapixels (2,400 × 864 pixels) and are equipped with 35 mm lenses. The tendon was illuminated by two LED lights (Schneider Optische Werke, GmbH, Bad Kreuznach, Germany). The DIC system was calibrated prior to the tests to accommodate a measurement volume of 235 × 170 × 160 mm. Images were collected using the ARAMIS DIC software (ARAMIS v2018, GOM, Braunschweig, Germany) at a frame rate between 32 and 384 Hz, with all specimens downsampled to 32 Hz during analysis. Image processing was performed using the ARAMIS DIC software using a subset size of 19 pixels, a step size of 12 pixels and a virtual strain gauge length of 2.2 mm.

### Histology

Samples (average 15 mm in width) were dissected from areas proximal and distal to the rupture site, fixed in 10 % neutral buffered formalin for 48 h, dehydrated through graded alcohol (70 %, 90 %, 95 %, 100 %, 100 %, 100 % ethanol) series and embedded in paraffin-wax. 5 μm-thick sections were rehydrated and stained with haematoxylin-eosin or alcian blue, pH 2.5. Stained sections were examined microscopically and assigned a Bonar score, a validated measure of tendon degeneration based upon cellularity, tenocyte shape, collagen structure, vascularity and ground substance (Maffulli et al., 2008). This provides an aggregate score between 0 (normal) and 12 (severely abnormal). Bright-field images were acquired using an automated inverted microscope (Olympus IX83). Images were acquired using a 20× magnification lens and the cell Sense Olympus imaging software.

### Data analysis

Primary outcome measurements were failure load, stiffness and engineering strain (*ε = Δl/lo*). Transverse strain (medio-lateral direction; *ε_x_*) and longitudinal strain (superior-inferior direction; *ε_y_*) were reported. Stiffness is defined as the resistance of the tendon to deform under load and was computed as the slope (slope = Δ force/Δ displacement) of the linear region of the force-displacement curve. This is the region of the curve between the toe region and the yield point. The toe region of the force-displacement curve is the non-linear region of the curve when the tendon is uncrimping or stretching out, and the yield point, also known as the failure region, is where the tendon is beyond its physiological limit, resulting in irreversible damage and plastic deformation. A custom-built Matlab (Mathworks, Natick, MA, USA) programme was used to determine the stiffness and yield point of the samples. This programme graphs the force *versus* displacement curves for each sample and requires the researcher to select the linear region of the graph. After completing this for each sample, the programme outputs both the stiffness and the yield point. Strain was obtained from the ARAMIS DIC software and its calculation of strain has been described in detail elsewhere (Luyckx et al., 2014; Zdero, 2017). Briefly, the software tracks the relative displacement between images of a stochastic pattern (speckling pattern) applied to the tendon’s surface during the loading cycle. It does this by matching subsets, which are known as a small group of pixels with unique grey values that are spaced by a specific step size. As mentioned above, a subset size of 19 pixels and a step size of 12 pixels were used for analysis. A strain tensor field was calculated from these images by converting pixels into absolute numerical values. The stereo camera set-up and the principles of triangulation enabled the calculation of precise coordinates of surfaces that were not completely flat. This allowed for accurately obtaining the strain magnitude and strain distribution of the entire surface. While this set-up could measure coordinates in three-dimensions, only data in two-dimensions were reported due to concerns over the accuracy of data in the third dimension, which is only valid if the volume of the specimen does not change under load. Each strain variable of interest was reported as the percent strain, which is the engineering strain multiplied by 100, and analysed by both studying its heat map and by averaging its value across the entire tendon surface and resampling it across a normalised load from the beginning of testing (0 % of failure load) to failure (100 % of failure load).

To analyse more closely the regional differences in strain during mechanical testing, tendons were divided into three regions (Fig. 1): distal, calcaneal insertion site to 2 cm proximal (region 1); mid-substance, 2-6 cm proximal from calcaneal insertion (region 2); proximal, > 6 cm proximal from calcaneal insertion to musculotendinous region (region 3).

Analysis of variance (ANOVA) tests were performed to determine significant differences between failure types for failure load and stiffness (*α* = 0.05).

## Results

### Mechanical testing

5 tendons failed in the mid-substance (region 2; Fig. 1) and 5 underwent calcaneus avulsions. 1 sample failed due to muscle tear before the tendon ruptured and was not included when performing ANOVA analysis of the mechanical testing data.

**Fig. 4.**
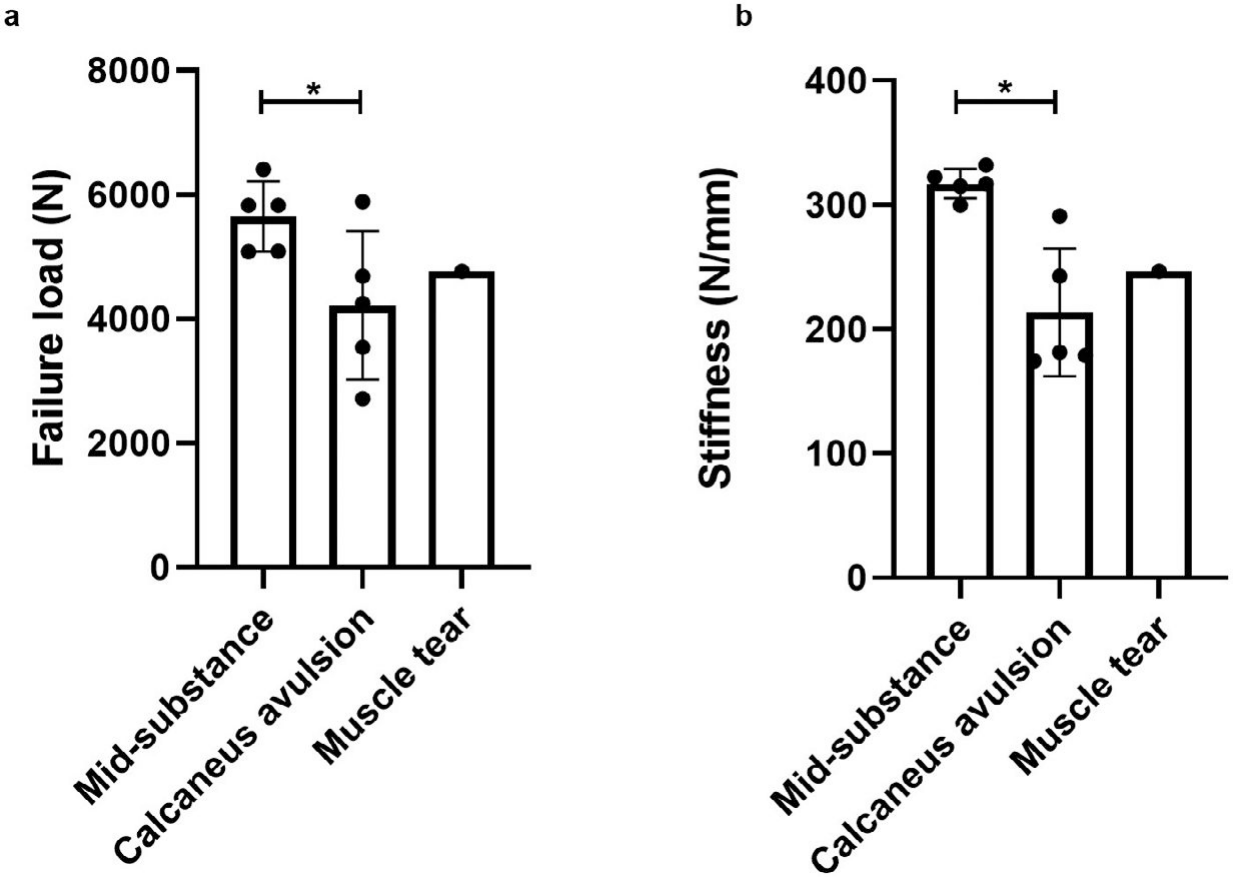
Mechanical testing results. Differences in (**a**) failure load and (**b**) tendon stiffness between the tendon failure types. * Significant differences (*p* < 0.05) were found between the mid-substance failures and calcaneus avulsion failures for failure load and tendon stiffness.

Mid-substance failures occurred at an average failure load of 5,649.1 ± 565.5 N (*n* = 5), while calcaneus avulsion failures (*n* = 5) occurred at a failure load of 4,216.9 ± 1,196.4 N (Fig. 4a; *p* = 0.04). Tendons that underwent calcaneus avulsions had significantly lower average tendon stiffness than those failing in the mid-substance (Fig. 3b; *p* = 0.002). There was no difference in the mechanical properties of tendons from male and female donors.

### Histology

**Fig. 5.**
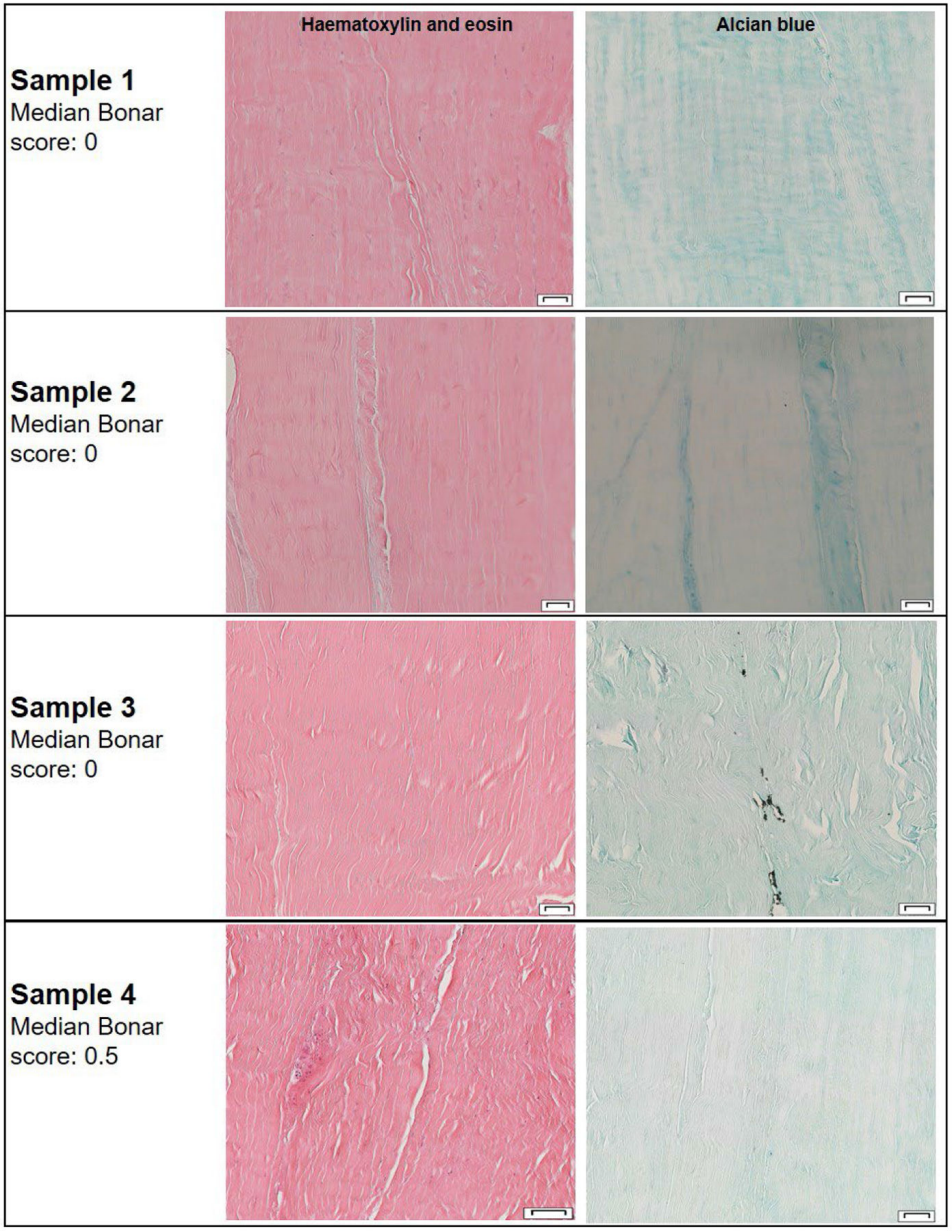
Representative histological sections of tendons stained with haematoxylin and eosin or alcian blue. Of note, histological images appeared normal, with no indication of prior disease of the tendon or breakdown of structural integrity. No specimen had a median Bonar score > 0.5. Scale bars: 50 μm.

Examination of the histological specimens provided Bonar scores of 0-0.5, in agreement with the gross anatomy observations, indicating that the tendons were without pre-existing tendinopathy. Representative histological images are shown in Fig. 5.

### Strain analysis

All 11 samples were included in the DIC assessment. Table 1 includes transverse and longitudinal strains at a percentage of failure load (25 %, 50 %, 75 % and 100 %) from each region for the tested Achilles tendon samples. These percentages corresponded to the linear region (25 % and 50 %), yield point (> 75 %) and failure region (100 %) of the load-deformation curve. As shown in Video 1, the deformation behaviour varied considerably from region to region within the tendon and also shifted dramatically as loading continued.

Early in the loading cycle, considerable transverse strain accumulated in the proximal region (region 3, Fig. 1) of the tendon, with little longitudinal or transverse strain apparent elsewhere. However, shortly before failure, significant longitudinal strain appeared for the first time in the location that would go on to rupture.

Fig. 5 shows the directional breakdown of strain in a representative Achilles tendon sample at the moment it sustained a mid-substance failure.

**Fig. 6.**
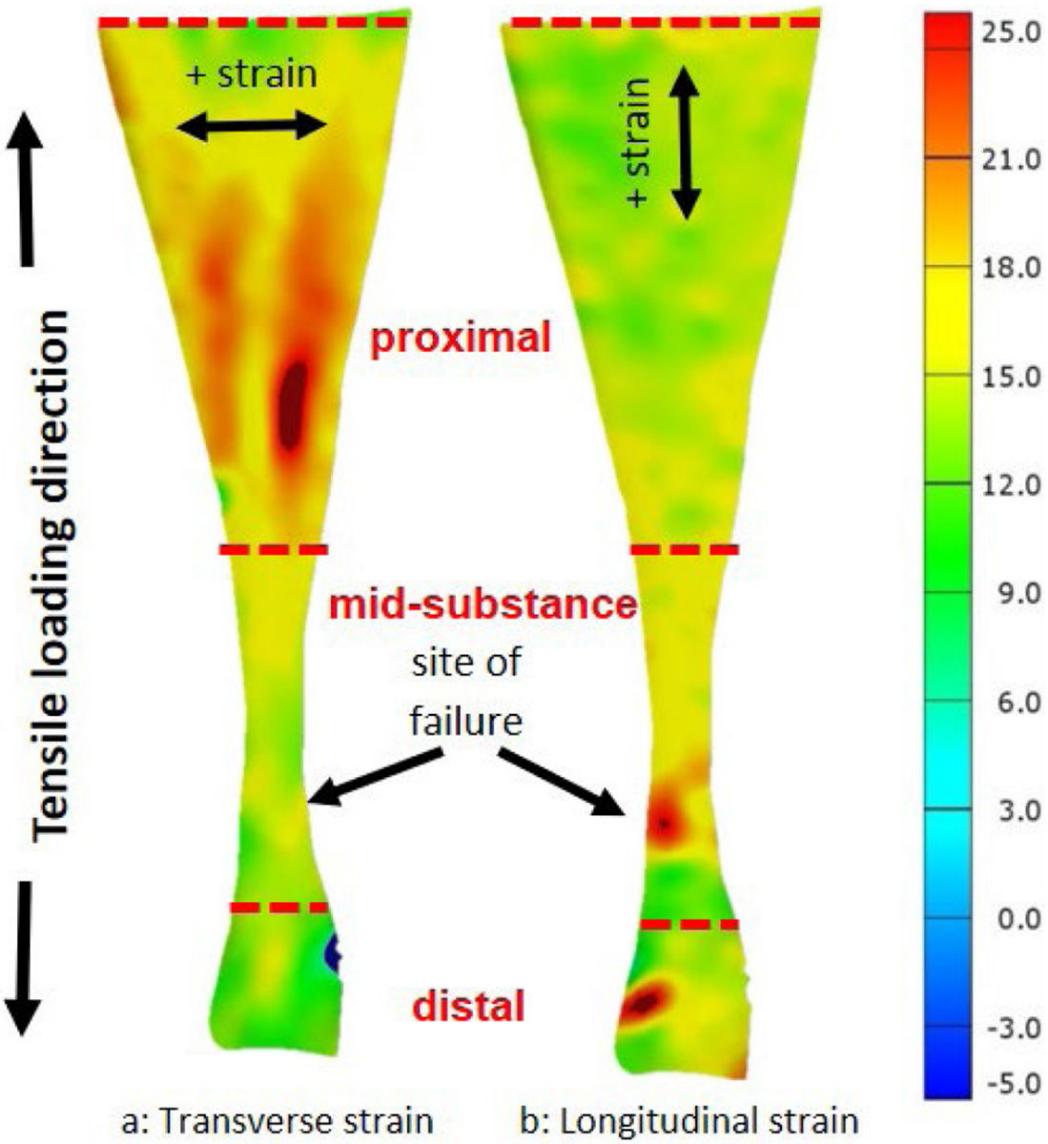
Directional breakdown of strain in one representative sample of a human Achilles tendon at rupture. At rupture, the tendons experienced greater (**a**) transverse strain than (**b**) longitudinal strain, in the proximal region (region 3), whereas the opposite is true at the rupture site (Video 1 shows evolution of strain in real time during loading to failure).

This observation was confirmed when the heat maps for the transverse strain (Fig. 6a) and longitudinal strain (Fig. 6b) were compared. It was striking how the tendons experienced transverse strain and that localised regional differences in strain patterns clearly existed. A strikingly consistent strain pattern was seen for all specimens, regardless of whether they failed in the mid-substance or by calcaneus avulsion. The pooled data are represented in Fig. 6, which shows the average longitudinal and transverse strains as a function of percent failure load for the entire surface and for regions 1, 2 and 3.

**Fig. 7.**
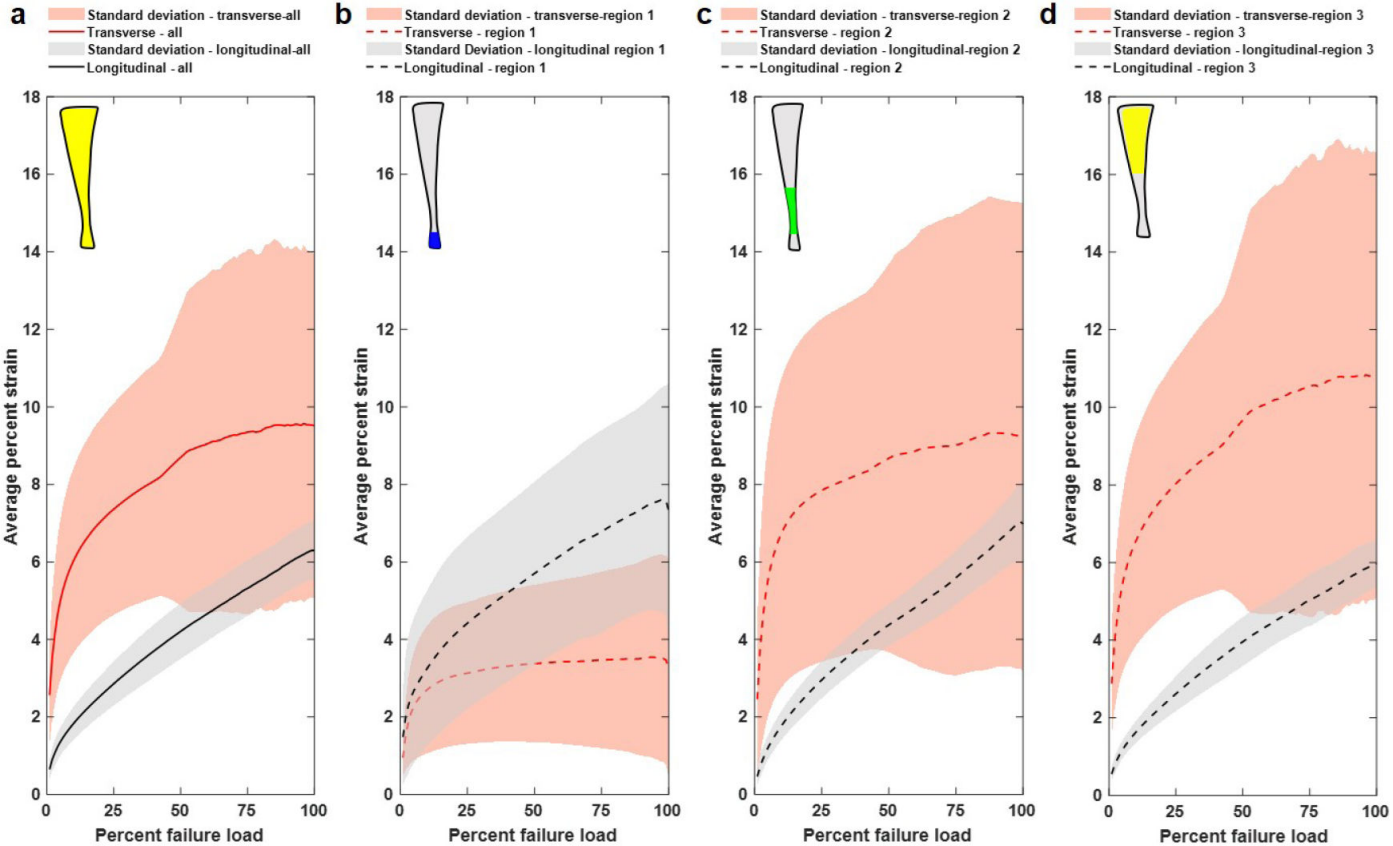
Strain behavior of the tendons during mechanical testing. Average percent transverse and longitudinal strain as a function of percent failure load for (**a**) the entire surface and (**b**) regions 1, (**c**) regions 2 and (**d**) regions 3. Under increasing load, regions 2 and 3 experience more transverse strain than longitudinal strain, which indicates that the samples are widening more than elongating under tensile stress. (**b**) Region 1 experienced more longitudinal strain than transverse strain throughout loading.

Overall, transverse strain increased dramatically as loading increased, with a plateau at an average value of 9.5 % strain. Average longitudinal strain increased more slowly, did not plateau and reached 6 % strain at the time of rupture (Fig. 7a). However, there were striking regional differences. Thus, in regions 2 (mid-substance) and 3 (proximal), transverse strain exceeded longitudinal strain throughout almost the entire tensile test, especially at low and medium loads (Fig. 7c,d). As loading increased, the transverse strain eventually plateaued, while longitudinal strain increased rapidly until failure. In region 1 (adjacent to the calcaneal insertion) the opposite was true and the longitudinal strain exceeded the transverse strain at all but the lowest loads (Fig. 7b).

## Discussion

In the present study, a failure testing was performed on relatively young, healthy, human Achilles tendons that, according to epidemiological studies, are less likely to sustain tendon ruptures. The values determined for the strength of the Achilles tendon were at the high end of those reported in the literature (Louis-Ugbo et al., 2004; Wren et al., 2003; Wren et al., 2001), consistent with the absence of pre-existing tendinopathy in the samples, as determined by careful gross inspection and histology.

DIC strain data showed that the high strains initially imposed by loading were almost entirely absorbed by the proximal region (region 3, Fig. 1) in the lateral direction, thereby shielding the mid-substance where the tendon is thinnest and most prone to rupture. Its ability to accommodate strain in the lateral direction was exhausted at approximately 99 % of the ultimate load of failure, at which point high longitudinal strain rapidly appeared for the first time in the mid-substance, which quickly snaped (see Video 1). These findings suggested that the remarkable tensile strength required to cause a mid-substance Achilles tendon rupture during *in vitro* mechanical testing may be associated with this unique deformation pattern of the tendon.

It remained unclear if these new insights into *in vitro* tendon failure mechanics are applicable to *in vivo* tendon ruptures, given the limitations of the *in vitro* mechanical testing. Further research is needed to understand how ageing and tendinopathy affect this behaviour as older individuals and people with tendinopathy are significantly more likely to sustain a tendon rupture. Luyckx et al. (2014) were the first to apply DIC to the human Achilles tendon. 6 tendons were loaded to 628.3 N, which, based upon the present study data, is only ~ 11 % of failure load. Similar to the current findings, they noted considerable regional inhomogeneity and also reported significant transverse strain.

The present study has certain limitations. It is important to note that the deformation patterns observed may be influenced by the *in vitro* mechanical testing set-up, which imposed boundary conditions that may propagate these complex loading patterns. The tendon was not directly clamped: the human Achilles tendons were secured by their natural insertion sites in the calcaneus and muscle. However, cryo-clamping the distal third of the *gastrocnemius* muscles and the myotendinous junctions of the tendons could contribute to the mechanical behaviour and complex strain behaviour observed. Specifically, compressing the muscle and tendinous junction could splay the tendon fibres and cause them to widen under tension. Additionally, because the muscle is unable to dissipate forces and absorb load, this creates an artificial boundary. It is possible that as the *gastrocnemius* and *soleus* muscle bear load, they will contract laterally, which would reduce the lateral movement of the myotendinous junction. The geometry of the tendon, which narrows in the mid-substance, creating wider insertions into the proximal muscle and distal calcaneus, may also contribute to this deformation response because, once stretched, the tendon fibres would strain laterally to straighten.

Another limitation of the present study was that strain and deformation in the anterior-posterior direction were not directly evaluated and shear stresses could not be measured because of the non-uniform cross-sectional area of the tendon. While the DIC system used was three-dimensional, its in-plane measurements were not sufficiently robust to compute strain directly in the anterior-posterior direction. Finally, a commercially available solid white, mineral-oil-based face paint was applied to the tendon’s surface minutes before testing. This material is not known to have any effects on the mechanics of the underlying tissue.

Further research is necessary to determine the molecular and structural basis for the unique strength of the healthy Achilles tendon (Docheva et al., 2015). In addition, it will be important to understand whether the localised regional differences in strain patterns are related to the bundles of collagen fascicles and the underlying biology of the tendon. *In vivo* evidence from Gatt et al. (2015) indicates widening of the tendon but it is not clear to what extent geometry or some intrinsic property of the tendon contributes to this behaviour. Studies that have tensile-tested the mid-substance of the Achilles tendon, thereby reducing the influence of the tendon’s geometry, have nevertheless noted significant transverse strains; however, this could be an artifact of the testing set-up (Luyckx et al., 2014).

The tendon has few cells and type I collagen makes up over 90 % of the extracellular matrix. This provides considerable tensile strength in the longitudinal direction but other components, possibly including fluid flow, presumably control transverse strain. It is interesting to note that the portion of the tendon closest to the calcaneus (region 1) displayed more longitudinal than transverse strain throughout loading. An alternative explanation may reside with the twist of the collagen fascicles that exists within the human Achilles tendon, which could produce the transverse strain. This will be explored in further studies; whether and how these structures are altered in tendinopathies may provide critical information.

In conclusion, using DIC to study the real-time strain behaviour of Achilles tendons as they are stretched to failure during *in vitro* mechanical testing, it was found that the tendons undergo large transverse strains prior to taking on longitudinal strains just before rupture. Interestingly, high longitudinal strains were not observed at the mid-substance failure site until just prior to rupture. Additionally, the study hypothesis was that the mechanical strength of uninjured Achilles tendons obtained from relatively young donors would be higher than those reported in literature. However, the average failure load for the mid-substance rupture cohort was at the high end of the values reported in the literature but did not exceed them. Future research will focus on determining whether the strain and mechanical behaviour observed when loading to failure is perturbed in older and tendinopathic Achilles tendon.

## Figures and Tables

**Video 1.** Achilles tendon rupture during in vitro mechanical testing. Real-time, deformation measurement of (**a**) percent transverse strain and (**b**) percent longitudinal strain as a human Achilles tendon is loaded to failure. The heat map scale on the right indicates strain from – 5 % (blue) to 25 % (red). As load is applied, percent strain initially accumulates in the widest region of the tendon (region 3; Fig. 1) in a transverse direction, with relatively little or no strain in the mid-substance (region 2; Fig. 1) where the tendon will eventually fail. As the tendon nears failure, the increasing percent transverse strain in the proximal region plateaus leading to a rapid increase in percent longitudinal strain in the tendon mid-substance which immediately ruptures. Video is available on journal website.

**Table 1.** Transverse and longitudinal strains as percentages of failure load in each region. Percent strain is determined by the multiplying the engineering strain by 100.

	Transverse strains											
	Region 1				Region 2				Region 3			
	Percent (%) failure load											
	25	50	75	100	25	50	75	100	25	50	75	100
**Calcaneus avulsion**	3.7	3.8	3.7	3	12.8	15.8	20.8	21.9	15	22	26.7	25.7
**Mid-substance tear**	3.2	3.7	3.8	2	4.9	5.9	6.6	5.5	8.5	9.8	11	12.3
**Calcaneus avulsion**	0.7	0.1	−0.6	−1.7	3.6	3	2.7	4	9.3	10.6	11.4	13.5
**Mid-substance tear**	1.9	2	2.1	1.7	3.7	3.7	3.7	4.9	5.9	6.7	7.3	7.5
**Calcaneus avulsion**	1.6	2.1	2.4	2.7	4.4	5.6	6.7	7.1	7.2	8.8	9.8	10.5
**Mid-substance tear**	5.1	5.2	5.2	5.6	9.4	9.4	6.3	6.5	8.6	9.4	9.8	10.3
**Mid-substance tear**	1.8	1.9	1.8	1.4	1.5	1.8	1.9	1.5	1.73	2.2	2.5	2.9
**Muscle failure**	1.5	1.9	2.2	2.5	7.9	9	8.4	8.2	6.1	7.6	6.8	6.6
**Calcaneus avulsion**	6.2	6.6	7	7.3	13.3	14	14.4	14.5	9	9.8	10.1	10
**Calcaneus avulsion**	5.4	6.3	7	8.2	13.4	13.1	12.9	12.4	9.2	10.8	11.4	11.5
**Mid-substance tear**	3.4	3.7	3.6	3.3	12.1	14.2	15	15	7.8	9.2	9.2	8.3
	Longitudinal strains											
	Region 1				Region 2				Region 3			
	Percent (%) failure load											
	25	50	75	100	25	50	75	100	25	50	75	100
**Calcaneus avulsion**	5.2	6.6	7.7	8.6	3.3	4.8	6.1	8	3.2	4.4	4.8	6
**Mid-substance tear**	3	4.4	5	2.5	2.7	4.2	5.7	6.2	2.6	4	5.1	6
**Calcaneus avulsion**	2.8	3.8	4.7	5.3	2.6	4	5.2	6.3	2	3.1	4.2	5.2
**Mid-substance tear**	2.5	3.6	4.6	4.1	3.7	5.1	6.4	7.6	2.9	4.3	5.4	6.5
**Calcaneus avulsion**	2.2	3.1	3.5	3.5	2.3	3.6	4.8	5.7	2.1	3.3	4.3	5.1
**Mid-substance tear**	6.8	8.6	10.3	11.4	3.2	4.4	4.9	7.7	2.6	3.8	4.9	6
**Mid-substance tear**	3.1	4.6	6.2	8.2	3	4.1	5	6	3	5	6.5	7.2
**Muscle failure**	4	4.9	6.1	7.3	2.2	3.7	5	6.2	1.9	3	4.4	5.4
**Calcaneus avulsion**	4	5.3	6.3	7.3	2.9	4.4	5.6	6.8	2.7	4	5	6
**Calcaneus avulsion**	9.6	10.9	12.2	13.2	3.3	4.7	5.9	7.1	3.1	4.5	5.4	5.8
**Mid-substance tear**	5.5	6.9	8.1	9.1	3.4	5.2	7	9.1	2.8	4.2	5.4	6.5
